# Spatiotemporal Controllability and Environmental Risk Assessment of Genetically Engineered Gene Drive Organisms from the Perspective of European Union Genetically Modified Organism Regulation

**DOI:** 10.1002/ieam.4278

**Published:** 2020-05-27

**Authors:** Christoph Then, Katharina Kawall, Nina Valenzuela

**Affiliations:** ^1^ Testbiotech e.V., Institute for Independent Impact Assessment of Biotechnology Munich Germany; ^2^ Fachstelle Gentechnik und Umwelt Munich Germany

**Keywords:** Genetically engineered gene drive organism, Environmental risk assessment, Next generation effects, Spatiotemporal control

## Abstract

Gene drive organisms are a recent development created by using methods of genetic engineering; they inherit genetic constructs that are passed on to future generations with a higher probability than with Mendelian inheritance. There are some specific challenges inherent to the environmental risk assessment (ERA) of genetically engineered (GE) gene drive organisms because subsequent generations of these GE organisms might show effects that were not observed or intended in the former generations. Unintended effects can emerge from interaction of the gene drive construct with the heterogeneous genetic background of natural populations and/or be triggered by changing environmental conditions. This is especially relevant in the case of gene drives with invasive characteristics and typically takes dozens of generations to render the desired effect. Under these circumstances, “next generation effects” can substantially increase the spatial and temporal complexity associated with a high level of uncertainty in ERA. To deal with these problems, we suggest the introduction of a new additional step in the ERA of GE gene drive organisms that takes 3 criteria into account: the biology of the target organisms, their naturally occurring interactions with the environment (biotic and abiotic), and their intended biological characteristics introduced by genetic engineering. These 3 criteria are merged to form an additional step in ERA, combining specific “knowns” and integrating areas of “known unknowns” and uncertainties, with the aim of assessing the spatiotemporal controllability of GE gene drive organisms. The establishment of assessing spatiotemporal controllability can be used to define so‐called “cut‐off criteria” in the risk analysis of GE gene drive organisms: If it is likely that GE gene drive organisms escape spatiotemporal controllability, the risk assessment cannot be sufficiently reliable because it is not conclusive. Under such circumstances, the environmental release of the GE gene drive organisms would not be compatible with the precautionary principle (PP). *Integr Environ Assess Manag* 2020;16:555–568. © 2020 The Authors. *Integrated Environmental Assessment and Management* published by Wiley Periodicals, Inc. on behalf of Society of Environmental Toxicology & Chemistry (SETAC)

## KEY POINTS

- We introduce the concept of spatiotemporal controllability of genetically engineered (GE) gene drive organisms: This approach uses specific knowns to decide upon known unknowns as a new step in the environmental risk assessment (ERA) of GE gene drive organisms.
- Three criteria are considered: the biology of the target organisms, their natural interactions with the environment, and their intended biological characteristics as introduced by GE.
- If there is a plausible risk that GE gene drive organisms can escape spatiotemporal controllability without effective means to control dispersal or persistence, a sufficiently reliable risk assessment will not be possible because it is not conclusive.
- We propose the spatiotemporal controllability as a cut‐off criterion in ERA of GE gene drive organisms: If a sufficiently reliable risk assessment is not possible in virtue of the absence of spatiotemporal controllability, the environmental release of the GE gene drive organism could not be allowed.

## INTRODUCTION

Genetically engineered (GE) organisms are not derived from natural evolutionary mechanisms and processes. Instead, genetic engineering technologies enable genetic rearrangements and disruptive additions of additional DNA coding for novel proteins or drastic metabolic changes, resulting from gene combinations that may be different compared to those stemming from conventional breeding or found in natural populations. Additionally, these genetic engineering techniques can cause specific unintended effects (see Forsbach et al. 2003; Kim et al. 2003; Makarevitch et al. 2003; Windels et al. 2003; Rang et al. 2005; Latham et al. 2006). Consequently, experience gained from, for example, conventional breeding cannot simply be extrapolated to the risk assessment of GE organisms. Many scientists emphasize the risks that releases of GE crop plants may carry if oversight is insufficient (see Quist and Chapela 2001; Piñeyro‐Nelson et al. 2009; Schafer et al. 2011; Wegier et al. 2011; Bauer‐Panskus et al. 2013; Lu et al. 2014; DeKeyser et al. 2015). Similar concerns have been raised in regard to GE fish, where survival, migration, spawning, hybridization, and introgression may occur under natural conditions and in different environments (Moreau et al. 2011; Sundström et al. 2014; Devos et al. 2019; Vandersteen et al. 2019). This is also the case in the field of synthetic biology, which covers a broad range of applications, where concerns have been raised about the consequences of a lack of control in regard to environmental releases (Tucker and Zilinskas 2006; Breckling and Schmidt 2015; Engelhard et al. 2016; Epstein and Vermeire 2016; Then 2016; Reeves and Phillipson 2017; Seager et al. 2017; Trump et al. 2017; Trump et al. 2018; Wang and Zhang 2019).

The need to retain control over releases of GE organisms is also reflected in international and national regulations in the European Union (EU) (see, e.g., Directive No 2001/18/EC [EC 2001]) or the Cartagena Protocol established under the Convention of Biological Diversity (CBD 2000).

More recently, genetic engineering techniques and applications have been developed to target natural populations rather than crop plants or farm animals. Most prominent are so‐called “gene drives” (Frieß et al. 2019). Besides gene drives, there are also other applications that target nondomesticated populations, including trees (Zhang et al. 2013), corals (Levin et al. 2017), fungi (Lovett et al. 2019), insects (Ant et al. 2012), viruses (Reeves et al. 2018), fish (Trump et al. 2018), or mammals (Redford et al. 2019).

Technical possibilities and consequent genetic engineering of nondomesticated populations can potentially interfere with evolutionary processes and therefore pose new challenges in risk management and risk assessment already discussed by others (Tucker and Zilinskas 2006; Oye et al. 2014; Breckling and Schmidt 2015; Reeves and Phillipson 2017; Trump et al. 2018; Wang and Zhang 2019). In the following sections, we discuss ways of dealing with these technical challenges, using gene drives as an example. These issues also raise ethical and societal questions that need to be discussed; these are not within the scope of the present paper and are discussed elsewhere (Schmidt et al. 2009; Bar‐Yam et al. 2012; Engelhard et al. 2016; Wolfe et al. 2016; Cummings and Kuzma 2017; Trump et al. 2018; CSS et al. 2019). The main focus of the present paper is environmental risk assessment (ERA) from the perspective of the precautionary principle (PP) as applied in the EU in regard to GE gene drive organisms. The PP plays a crucial role in EU regulation to enable decisions on risks against a background of uncertainties. It is one of the fundamental principles of EU policy in order to protect the environment, health, and food safety (EC 2000). The PP was formally adopted in the Maastricht Treaty in 1992 (EU 1992), it is enshrined in Article 191(2) of the Treaty on the Functioning of the European Union (EU 2012) and has been incorporated into a number of secondary legislation measures (regulations and directives), which apply to their member states. The main feature of the PP is the prevention of risks in the face of scientific uncertainty, aiming to avoid harm before a hazard manifests (for more details, see Fisher et al. 2006; Garnett and Parsons 2017). In this context, it is important that the PP is of fundamental significance for genetically modified organism (GMO) regulation in the EU (Article 1 in EC 2001). Based on the PP, the EU GMO regulation requests that uncertainties and the boundaries of knowledge in risk assessment and risk management of GE organisms (see Böschen 2009) are considered. If an adequate management of uncertainties and nonknowledge cannot be established, the EU GMO regulation cannot be implemented. The present paper firstly provides a brief overview of the technical background of GE gene drive organisms and identifies specific challenges for the ERA of these organisms. Then we discuss spatiotemporal controllability as a cut‐off criterion and discuss the use of certain criteria in the ERA of chemicals, pesticides, and GE gene drive organisms. In a case study, we exemplify how these criteria can be applied. Lastly, we discuss how these criteria can be integrated into the current EU GMO regulation.

## TECHNICAL BACKGROUND: GE GENE DRIVES

Genetically engineered gene drive organisms are designed to introduce artificial genetic elements into natural populations. Subsequent sexual reproduction ensures that the organisms produce offspring intended to spread and propagate GE traits beyond the Mendelian pattern of inheritance throughout wild populations (Windbichler et al. 2011; Gantz et al. 2015).

Most GE gene drives currently being developed are intended for release into natural populations to decrease the spread of insect‐borne diseases or to reduce agricultural and environmental damage caused by pests. Currently, there are 2 basic GE gene drive concepts: “Suppression drives” are meant to introduce genetic elements that reduce or eradicate natural populations, for example, by interfering with their capacity to reproduce (Kyrou et al. 2018); “replacement drives” are meant to replace natural populations with persistent GE populations with altered biological characteristics, inheriting artificial genetic elements (Gantz et al. 2015).

Naturally occurring selfish genetic elements, such as transposable elements, meiotic drive genes, and homing endonuclease genes, may be used to generate synthetic gene drives and can be adapted to their respective requirements (Sinkins and Gould 2006).

Several genetic engineering techniques can be used to create a gene drive. The present paper focuses on gene drives that are based on Clustered Regularly Interspaced Short Palindromic Repeat/CRISPR‐associated protein (CRISPR/Cas) even though other kinds of gene drives exist, and our conclusions are not limited to this specific technique. The development of the CRISPR/Cas system has revolutionized biotechnology in recent years (Jinek et al. 2012) and has already been used to alter the genomes of many plant and animal species (Jiang et al. 2013; Jinek et al. 2013; Li et al. 2013; Bassett and Liu 2014; Kistler et al. 2015).

The CRISPR systems originate from an adaptive immune system of bacteria and archaea (Mojica et al. 2005; Barrangou et al. 2007). The biotechnology tool CRISPR/Cas allows the precise targeting of an endonuclease (e.g., Cas9 derived from *Streptococcus pyogenes*) to specific genomic regions via a guide RNA (gRNA) (Jinek et al. 2012; Doudna and Charpentier 2014). The Cas introduces a DNA double strand break at the target site, which subsequently activates cellular repair mechanisms: The break can either be repaired due to an error‐prone repair mechanism introducing random changes at the target site or according to the sequence of a DNA template if provided. A more detailed description of the CRISPR/Cas mechanism is discussed in‐depth elsewhere (Wang et al. 2016; Jiang and Doudna 2017).

The CRISPR/Cas‐based GE gene drive organisms replicate the process of genetic engineering in a self‐organized way: In each generation, the offspring receive 1 chromosome carrying the genetic material encoding the CRISPR/Cas components (e.g., the nuclease Cas9 and a gRNA) and the associated cargo‐genes (altogether called the “gene drive cassette”). When both the Cas nuclease and the gRNA are expressed in the germline, the entire gene drive cassette is inserted at the target site determined by the gRNA, generating a homozygous organism in regard to the gene drive cassette.

Thus, all progeny in the next generations will have the gene drive cassette. As a result, once introduced, the gene drive cassette can spread throughout a population much more rapidly than could be expected with the Mendelian pattern of inheritance. This process was rightfully named “mutagenic chain reaction” because it autocatalytically produces homozygous mutations (Gantz and Bier 2015; Ledford 2016).

Gene drives based on CRISPR/Cas have thus far been implemented in yeast (DiCarlo et al. 2015), mosquitoes (Hammond et al. 2016; Kyrou et al. 2018), flies (Gantz and Bier 2015; Buchman et al. 2018), and mice (Grunwald et al. 2019).

## SPECIFIC CHALLENGES IN THE ERA OF GE GENE DRIVE ORGANISMS

If there is an application for the release, the ERA of GE gene drive organism needs to consider uncertainties and limits of knowledge in at least 3 different areas: the technology, the target organism, and the receiving environment, including nontarget organisms (Oye et al. 2014; Akbari et al. 2015; Kuzma et al. 2018; Noble et al. 2018).

According to EU regulation (EC 2001), all organisms derived from genetic engineering processes require risk assessment before they can be released into the environment. This also applies to gene drive organisms derived from methods of genetic engineering as described in the *Introduction* and *Technical Background* sections. However, there are major differences that need to be taken into account to distinguish GE gene drive organisms from GE organisms already assessed by EU institutions. A newly released gene drive will typically take dozens of generations to affect a substantial proportion of a target population (Oye et al. 2014). Therefore, issues such as genetic stability will require more attention in ERA of GE gene drive organisms compared to crop plants with seeds propagated under controlled conditions by a company. Unlike with GE organisms, there is no practical experience with the ERA of GE gene drives, but there are new challenges and both need to be considered when defining problem formulations for their ERA (Simon et al. 2018). Table 1 provides an overview of some of these differences, which may be summarized as “next generation effects,” emerging from interactions with complex environments and the heterogeneous genetic backgrounds in natural populations (Bauer‐Panskus et al. 2020).

**Table 1:** New challenges in the ERA of GE gene drive organisms in comparison to experience with GE crop plants

Assumptions in the risk assessment of GE crop plants	New challenges in ERA of GE gene drive organisms
The majority of crop plants are cultivated for a single growing period. These plants are not meant to reproduce outside cultivation.	Next generations will emerge spontaneously; the process of genetic engineering is a self‐organized process replicating in each generation.
Due to previous breeding processes, plant varieties used for genetic engineering are relatively stable and have defined characteristics, as well as a reduced genetic diversity. Seed quality can be controlled by breeders (or farmers) before and during cultivation.	Wild populations very often contain a broad spectrum of genetic backgrounds. As a result, GE gene drive organisms introduce their new genetic information into heterogeneous genetic backgrounds without additional controls in place, such as those used in the laboratory or by the breeder.
Crop plants are often grown in a managed agricultural environment with reduced biodiversity.	Wild populations very often interact with complex ecosystems.
Crop plants of the same species are often cultivated under similar environmental conditions.	Wild populations, e.g., insects are often exposed to a wider range of environmental conditions due to their mobility. Further impact factors include, e.g., seasonal changes.

ERA = environmental risk assessment; GE = genetically engineered.

Genetically engineered crops typically are not meant for spontaneous propagation or to spread in the environment. Nevertheless, there are several cases of cultivation‐independent establishment of GE plants (Bauer‐Panskus et al. 2013), which can help to identify new challenges for the ERA of GE gene drive organisms, such as categorized in Table 1. We identified several publications dealing with unintended next generation effects that were not observed or intended in the original GE event (Cao et al. 2009; Kawata et al. 2009; Yang et al. 2017). Unintended effects can also emerge from interaction of the newly inserted genes with different genetic backgrounds (Adamczyk and Meredith 2004; Vacher et al. 2004; Adamczyk et al. 2008; Lu and Yang 2009; Bollinedi et al. 2017). Furthermore, unintended genomic effects can be triggered by changing environmental conditions or biotic and abiotic stressors (Meyer et al. 1992; Matthews et al. 2005; Then and Lorch 2008; Zeller et al. 2010; Trtikova et al. 2015; Fang et al. 2018; Zhu et al. 2018). Concerns being raised by scientists working in the field of synthetic biology are similar to those in regard to effects emerging from the interaction of synthetic organisms, which are either generated by modifying existing biological systems and/or recreated with the environment and/or with specific genetic backgrounds (see Tucker and Zilinskas 2006; Breckling and Schmidt 2015; Epstein and Vermeire 2016; Seager et al. 2017; Trump et al. 2018; Wang and Zhang 2019).

Thus, under conditions of self‐organization, self‐reproduction, and interactions with environmental stressors, next generation effects can occur in GE organisms that cannot be predicted from previous generations (Bauer‐Panskus et al. 2020). This is especially relevant for the ERA of those gene drive organisms that 1) depend on the process of spontaneous self‐reproduction, 2) will be exposed to a high range of genetic diversity within the target populations, and 3) are likely to interact with complex environments.

These findings are also reflected in the guidance published by the European Food Safety Authority (EFSA) in 2013, dealing with the ERA of GE animals, including GE insects and mentioning GE gene drive organisms (EFSA 2013a). So far, this guidance has not been used because there are no applications for the release of GE animals in the EU. Nevertheless, EFSA's guidance on the ERA of GE animals is relevant in this context, given that globally, it is the first guidance of a competent authority which covers, to some extent, the ERA of GE gene drive organisms and of other GE organisms that can spontaneously propagate in the environment.

In this guidance EFSA raises questions about the impact of the genetic background in natural populations where recombinant DNA is introduced into genetic backgrounds of not only domesticated plants and animals, but also those of wild populations (EFSA 2013a). Furthermore, according to EFSA, the whole life cycle of the GE animal needs to be considered because the receiving environments might determine possible adverse effects over time. In addition, EFSA explains that long‐term effects may occur due to increases in spatial and temporal complexity: “i. The ecological functions of specific species and their complex biotic or abiotic interactions (…) are not always fully understood. ii. The methodologies for testing potential effects on NTOs (non‐target organisms) are limited. Field trials might not be feasible in all cases, as it might be impossible to eradicate the released GM insect population if an adverse effect is identified related to the release, in particular, applying replacement strategies. iii. The fact that it is not feasible to simulate the complexity of the receiving environments in laboratory tests, semi‐field tests or modelling. (…) Consequences of the decrease or eradication in population size of a certain species or the replacement of wild population by GM insect populations might not be predictable.” (EFSA 2013a, p 103)

Interestingly, in 2018, the European Commission requested EFSA to evaluate whether the current ERA guidelines were still suitable for the ERA of GE gene drive organisms (EFSA 2018). The final opinion on this issue is expected from EFSA in 2020.

Our comparison of GE crops and GE gene drive organisms (Table 1), which is supported by reviewed literature on GE crops, EFSA's guidance on the ERA of GE animals (EFSA 2013a), and other research by scientists in the field of synthetic biology, shows that GE organisms carrying a gene drive do indeed pose new challenges to current EU risk assessment. In short, “next generation effects” will substantially increase the spatial and temporal complexity that should be taken into account within ERA. In the light of these findings, the following questions are crucial for assessing the spatial and temporal complexity related to the release of GE gene drive organisms:

1) Can the genetic stability of the GE organism's introduced trait be controlled in following generations?
2) How can the genetic diversity of the target population be taken into account in risk assessment?
3) Is there a potential for gene flow to other species?
4) How can population dynamics and life cycle aspects of the target species be integrated in risk considerations?
5) Can the receiving environment be defined in regard to relevant interactions and confined in regard to potential spread?

Table 2 explains the relevance of these questions for the ERA of GE gene drive organisms and also discusses whether the questions are testable using available scientific methods. It indicates that in many cases significant uncertainties remain and some unknowns might prevail, making the risk assessment inconclusive. Especially the necessity of thoroughly assessing subsequent generations poses a big challenge: Although genetic stability over several generations and also the impact of some genetic backgrounds might be demonstrated in the laboratory, genome × environmental interactions and introgression into untested heterogeneous genetic backgrounds can still trigger unpredictable next generation effects.

**Table 2:** Overview of relevant questions for the ERA of GE gene drive organisms in terms of spatial and temporal complexity

Question	Relevance	Which methodology is available?
1) Can genetic stability be controlled in following generations?	Self‐replication and environmental as well as epigenetic effects can lead to emergence of next generation effects not observed in the first generation.	Several generations should be observed under controlled conditions applying a wide range of defined environmental conditions, which allows the assessment of at least short‐term evolutionary effects. The outcome has to be put in context to questions 2 and 3.
2) How can genetic diversity in the target population be taken into account?	In most cases, a high degree of genetic diversity exists in natural populations. These heterogeneous, genetic backgrounds can trigger unexpected effects not observed in lab populations.	In most cases, the inserted genes cannot be tested in interaction with the genetic diversity within natural populations. For example, in insects, the strains reared in the lab might represent only a small selection of the genetic diversity within wild populations.
3) Will there be any gene flow to other species?	If gene flow is possible and hybrid offspring are viable, the resulting organisms have to be seen as new events that need to be assessed separately from the original GE organisms.	It might be possible to perform hybridization experiments under controlled conditions. Results have to be put in context with questions 1 and 2.
4) How can population dynamics and life cycle aspects of the target species be integrated?	Bottlenecks in the population dynamics, e.g., due to the winter season, might result in inbreeding and changes in genetic variability. Bottlenecks can have a significant impact on tipping points within the population dynamics.	Large‐scale population effects can be modeled, but empirical investigations are difficult. Further, any results have to be interpreted in the light of questions 1 and 2.
5) Can the receiving environment be defined in regard to relevant interactions and confined in regard to potential spread?	Adverse effects can emerge from interaction with different components of the environment (such as associated microbiomes, symbionts, food webs, predators). Terrestrial and aquatic systems have to be taken into account, as well as complex interrelations (such as signaling pathways) and behavioral aspects. Interrelations may vary greatly throughout the life cycle (different developmental stages such as egg, larva, pupa, adult).	These aspects have to be assessed case by case and step by step. In most cases, long‐term, cumulative, and combinatorial effects cannot be tested or investigated ex ante.

ERA = environmental risk assessment; GE = genetically engineered.

## SPATIOTEMPORAL CONTROLLABILITY AS A CUT‐OFF CRITERION

The increase in spatial and temporal complexity associated with the release of GE gene drive organisms is likely to decrease the robustness of the ERA, especially if several generations are involved. If the persistence of a GE gene drive organism cannot be delimited in terms of time and space, its ERA has to consider long‐term dimensions, for example, by addressing the alteration of its gene drive mechanism under evolutionary pressure. Evolutionary processes make it possible to turn events with a low probability of ever happening into events that are likely to happen (Breckling 2013). Inherent nonknowledge can, thereby, increase to such an extent that the conclusiveness of risk assessment is severely affected. The key questions are: How can nonknowledge (see Böschen 2009), uncertainties, or as EFSA (EFSA 2013a) puts it, “incertitude, caused by limitations of scientific knowledge and knowledge production systems,” be integrated into a regulatory system of decision making? How is it possible to provide sufficient knowledge to facilitate reliable decision making in the ERA of GE gene drive organisms? We propose answering these questions by following a similar approach to the one established in the EU regulation of chemicals (Regulation (EC) No 1907/2006 [EC 2006]) and pesticides (Regulation (EC) No 1107/2009 [EC 2009]).

### Cut‐off criteria in the EU regulation of chemicals and pesticides

Spatial and temporal complexity plays a decisive role in the EU risk assessment of chemical substances. Recital 76 of Regulation (EC) No 1907/2006 (EC 2006) concerning the Registration, Evaluation, Authorisation and Restriction of Chemicals (REACH) addresses the spatial and temporal dimension: “Experience at international level shows that substances with characteristics rendering them persistent, likely to bio‐accumulate and toxic, or very persistent and very likely to bio‐accumulate, present a very high concern, while criteria have been developed allowing the identification of such substances.” Consequently, specific criteria to identify persistent, bioaccumulative, and toxic (PBT), as well as very persistent and very bioaccumulative (vPvB) chemical substances, are defined in ANNEX XIII of Regulation (EC) No 1907/2006 (EC 2006) and also in Annex II of Regulation (EC) No 1107/2009 (EC 2009) concerning the placing of plant protection products on the market. In particular, Regulation (EC) No 1107/2009 (EC 2009) integrates the criteria of persistent organic pollutant (POP), PBT, and vPvB substances into the regulatory decision‐making process. These criteria function as so‐called “cut‐off criteria,” which determine that the approval process should not proceed if a particular substance is classified as POP, PBT, or vPvB. In this context, it is important that the chemical substances are not only assessed in regard to their toxicity but also, more generally, in regard to their fate and behavior in the environment (Annex II, 3.7 of EC 2009), which gives decisive weight to the spatial and temporal dimension. If a substance is regarded as vPvB, there might still be some uncertainty or nonknowledge in regard to its actual long‐term adverse effects. Therefore, according to Regulation (EC) No 1107/2009 (EC 2009), it cannot be approved. For example, Annex II, point 3.7.3 (EC 2009) determines that an active substance, safener, or synergist will be approved only if it is not considered to be a vPvB substance.

### Cut‐off criteria in the ERA of GE gene drive organisms

The way in which cut‐off criteria were established for chemicals can serve as a useful model and be adapted for the risk assessment of GE organisms, especially for GE gene drive organisms. Similarly to EU regulation of chemicals (EC 2006), the fate and behavior of the organisms in the environment should be a crucial aspect. If there is a plausible risk that GE gene drive organisms can escape spatiotemporal controllability without effective means to control dispersal or persistence, then the authorization process could not proceed and the environmental release of the GE gene drive organisms would not be allowed.

In the context of chemical substances, cut‐off criteria should be well defined. Well‐known characteristics of the substances are used to integrate uncertainties around actual long‐term impacts into decision making. Similarly, the criteria applied in the ERA of GE gene drive organisms should be entirely clear and well defined. Therefore, we propose applying 3 well‐established scientific criteria:

1) the (natural) biology of the target organisms,
2) their (naturally) occurring interactions with the environment (biotic and abiotic), and
3) the intended biological characteristics of the organisms introduced by methods of genetic engineering.

These criteria should be combined in the ERA of GE gene drive organisms, with the aim of evaluating spatiotemporal controllability. Table 3 provides an overview of the most relevant details that can be used to evaluate spatiotemporal controllability in these 3 criteria.

**Table 3:** The main scientific criteria and their subsequent aspects for ERA of GE gene drive organisms in terms of spatiotemporal controllability^a^

Biology of the target species (wild type)	Interactions of the target species (wild type) with the environment	Intended biological characteristics of the GE organism
Potential to persist and propagate	Interactions within the ecosystem: position in the food web closely associated organisms (microbiome, parasites, symbiotic organisms) within the wider environment (beneficial insects, soil organisms, protected species)	Is the GE organism intended to produce more than 1 generation after release?
Population dynamics and life cycle	Role and function in energy and nutrient cycles	How can genetic stability be controlled in following generations after the release?
Potential to spread beyond fields and/or into different ecosystems	Impact of biotic stressors, e.g., pests and pathogens (whole life cycle)	Does the trait impact the fitness of the organisms?
Potential for reproduction with wild populations of the target species; genetic diversity in wild populations	Occurrence of abiotic stressors such as climate conditions (whole life cycle)	Does the trait impact the composition of biologically active compounds?
Potential for gene flow to other species		Can the persistence of the organisms be determined if necessary?

ERA = environmental risk assessment; GE = genetically engineered.

^a^ Vertical reading; aspects in each row are not specifically linked to each other; each column stands alone.

### Case study: How to apply the concept of spatiotemporal controllability

The following case study exemplifies how the concept of spatiotemporal controllability (Table 3) can be applied in ERA; it is a hypothetical case study of a GE olive fly (*Bactrocera oleae*) that contains a CRISPR/Cas‐based gene drive. As yet, there have been no reports of olive flies that are genetically engineered to carry a CRISPR/Cas‐based gene drive. Nevertheless, we used the olive fly as a model to analyze the spatiotemporal controllability of a GE gene drive organism because this fly is a relevant agricultural pest in Europe, especially in the Mediterranean area where it causes heavy losses in olive cultivation. We chose this example over other cases, such as the malaria vector *Anopheles*, because we wanted to investigate an organism that might become the first GE gene drive organism in Europe. Olive flies have previously been genetically engineered using “release of insects carrying a dominant lethal genetic system” (RIDL) technology, which was developed by the company Oxitec and has already been used in *Aedes* mosquitoes and olive flies (*B. oleae*) (Ant et al. 2012; Alphey et al. 2013). Insects genetically engineered with RIDL technology carry a conditional lethality trait that is inherited according to Mendelian laws. The effects emerging from olive flies carrying the transgene are gender specific: The male offspring will survive; the female offspring will die at the larval stage. As a result, the natural population of olive flies will supposedly decrease (for more background, see Ant et al. 2012). Olive flies already were applied for caged field trials in the EU in 2013 and 2015, although withdrawn due to political disputes and time delays (Schwindenhammer 2020).

In our case study (Table 4), we hypothetically investigate a CRISPR/Cas‐based gene drive in olive flies that causes female lethality during their embryonic development; the fertile male offspring further propagate the drive in accordance with the properties inherited by Oxitec's olive flies genetically engineered with RIDL‐technology (Ant et al. 2012). The olive flies in the case study are genetically engineered with a CRISPR/Cas‐based gene drive that converts the germline cells, which are originally heterozygous for the artificial gene construct, into homozygous cells, resulting in super‐Mendelian inheritance. Thus, the gene drive is capable of rapidly spreading through wild olive fly populations. Environmental factors can, however, lead to genetic changes in the GE gene drive olive flies, which can result in unforeseen and unwanted properties in subsequent generations. Mathematical modeling showed that CRISPR/Cas‐based gene drive systems are likely to be invasive, resulting in a very efficient spread of the gene drive in wild populations (Noble et al. 2018; Frieß et al. 2019). Many publications about GE gene drives show they are as yet still prone to the rapid evolution of resistance to the gene drive (Unckless et al. 2016; Champer et al. 2017; KaramiNejadRanjbar et al. 2018). Nevertheless, even a small number of GE gene drive organisms that reach only a modest fraction of the population is likely to be invasive (Noble et al. 2018).

**Table 4:** Example of spatiotemporal controllability assessment for hypothetical experimental field trials of GE gene drive olive flies in Spain^a^

Biology of the target species	Interactions with the environment	Intended biological characteristics of the GE organism
Olive flies are a wild species that can persist and propagate in the Mediterranean area and in regions with a similar climate. Their habitat is not clearly confined, except for the presence of olive trees (Nardi et al. 2005; Daane and Johnson 2010). Under specific conditions, such as high population densities, maximum dispersal distances for olive flies range from 4000 to 5000 m (Remund et al. 1976; Economopoulos et al. 1978).	There are complex interactions with other species such as birds, spiders, ants, chalcid wasps, and symbiotic bacteria (Neuenschwander et al. 1983; Bigler et al. 1986; Daane and Johnson 2010; Gonçalves et al. 2012; Picchi et al. 2016). The interrelationships include grazing, predation, and symbiosis. The interrelations vary greatly throughout the life history of the flies and different developmental stages (egg, larva, pupa, adult).	The trait is unlikely to enhance fitness; however, the gene drive is capable of spreading through wild olive fly populations, resulting in female lethality but fertile male offspring that further propagate the drive.
Population dynamics and life cycle go through several stages (egg, larva, pupa, adult) and are subjected to winter seasons, creating potential bottlenecks in regional populations (Ochando and Reyes 2000; Augustinos et al. 2005).	There are specific and symbiotic microbes associated with the olive flies (Capuzzo 2005; Ben‐Yosef et al. 2014).	Once released, the GE flies will mate in natural populations and cause the emergence of next generations without human intervention. Next generation effects might occur without being noticed.
Molecular analyses indicate a high level of gene flow among the Mediterranean populations (Ochando and Reyes 2000; Augustinos et al. 2005; Segura et al. 2008).		If the population is suppressed to a certain degree, it may be assumed that, depending on the amount and frequency of GE flies released, they might be eliminated after a period of time. However, various factors can have an impact on these processes, and their actual duration cannot be determined.
There are other known species that can mate with olive flies. However, it is unclear whether they can produce viable offspring and enable gene flow (Schutze et al. 2013).		

GE = genetically engineered.

^a^ Vertical reading; aspects in each row are not specifically linked to each other; each column stands alone.

Our spatiotemporal controllability assessment for the hypothetical case GE gene drive olive flies (see Table 4) shows that, based on the data available, spatiotemporal controllability is not given. Therefore, the approval process may proceed only if the olive flies are kept in the laboratory in isolated regions where no native populations of olive flies occur, or kept in a laboratory with high biosafety standards that specifically regulate the containment of GE gene drive organisms (Akbari et al. 2015).

In other cases the outcome of assessing spatiotemporal controllability might be different: Many researchers currently developing gene drive applications are already aware of the problem of spatial and temporal complexity (Noble et al. 2018). At present, several projects are developing gene drives that will be refined to specific regions or defined periods of time; for example, daisy‐chain drives were developed that are a self‐exhausting form of CRISPR‐based gene drives (Noble et al. 2019). Daisy drives create a cascade of drive alleles working together as a daisy chain. The daisy elements are inherited under Mendelian laws, leading to a disruption of the gene drive spread after a couple of generations if the offspring receive no daisy element. So far, mathematical models have been developed suggesting that daisy‐chain drives will not spread indefinitely through the target population (Noble et al. 2019). Researchers are also developing local gene drives that employ locally fixed alleles as the target for a gene drive on a particular island, assuming efficient genetic isolation from neighboring populations (Sudweeks et al. 2019). These technical approaches for assuring spatiotemporal controllability will require in‐depth analysis in regard to their de facto reliability and predictability. However, we do not exclude that, in the future, gene drives might become available which allow higher standards in regard to spatiotemporal controllability if compared to the olive fly case study.

In summary, there are no apparent obstacles to the application of spatiotemporal controllability assessment for GE gene drive organisms. Therefore, meaningful results and case‐specific outcomes can be expected from this future assessment methodology. If the criterion of spatiotemporal controllability were to be applied in the assessment of GE crop plants (see Bauer‐Panskus et al. 2013; Trtikova et al. 2017; Ellstrand 2018) or organisms derived from synthetic biology (see Epstein and Vermeire 2016; Trump et al. 2018; Wang and Zhang 2019), the outcomes are likely to differ from case to case.

## HOW TO INTEGRATE THE CRITERION OF SPATIOTEMPORAL CONTROLLABILITY INTO CURRENT EU GMO REGULATION?

In the EU, the regulatory system for GE organisms is based on a system of risk analysis set out in Regulation (EC) No 178/2002: Risk analysis (EC 2002) is particularly based on risk assessment, which is carried out by the EFSA, and risk management performed by the European Commission and the EU member states. Additional regulations concern specific aspects such as environmental releases (EC 2001) and food and feed safety (EC 2003). Final decision making in the EU approval process resides with the risk manager and in risk assessment policy (Millstone et al. 2008).

In this context, the risk manager, and especially the European Commission, can set adequate standards in risk assessment by establishing a robust framework for EFSA (Millstone et al. 2008). It is interesting to note that the European Commission adopted Regulation (EU) No 503/2013 (EC 2013), which sets the standards for assessing food and feed safety. However, no similar implementing regulation has yet been set out by the European Commission for the ERA of GE organisms. Furthermore, the integration of the concept of spatiotemporal controllability does not require a change in EU GMO regulation. The EU Directive 2001/18/EC (EC 2001) can be used as a legal basis to set relevant standards: According to Krämer (2013), spatial and temporal control is a necessary prerequisite to enable the PP. He concludes that if there is a likelihood that genetically modified plants or animals cannot be retrieved, the legal obligation to ensure that any release must be “safe” requires the refusal to authorize such releases (Krämer 2013). Thus, without having to change the overall legal framework, the European Commission could request EFSA to assess spatiotemporal controllability to deal with substantial uncertainties and nonknowledge as a matter of implementing the existing regulations.

Importantly, the assessment of spatiotemporal controllability as suggested is not an assessment of specific risk per se. Rather, it is related to the overall conclusiveness of the risk assessment. If EFSA comes to the conclusion that spatiotemporal controllability cannot be demonstrated, the risk assessment cannot be concluded. As a consequence, the overall approval process would be postponed or terminated by the risk manager. There are already several examples of inconclusive EFSA opinions that halted or substantially delayed the approval process, for example, EFSA opinions on maize 98140 (EFSA 2013b) and maize 3272 (EFSA 2013c). In an EFSA presentation from 2018, the following reasons were given for rendering scientific opinions of EFSA inconclusive: 1) “lack of sufficient data to conclude the risk assessment”, 2) “lack of toxicological study”, 3) “incomplete set of data linked to genotoxicity”, 4) “lack of complete set of compositional data”, 5) “lack of data to characterize the process / the product”, 6) “lack of data on efficacy”, 7) “waiving of data”, and 8) “inadequate study design” (Waigmann 2018).

## DISCUSSION

The development of GE gene drive organisms creates new challenges in ERA. Some of them also concern the risk assessment of GE crops (see, e.g., Bauer‐Panskus et al. 2013) or organisms derived from synthetic biology (Tucker and Zilinskas 2006; Breckling and Schmidt 2015; Epstein and Vermeire 2016; Seager et al. 2017; Trump et al. 2018; Wang and Zhang 2019). In general, if GE organisms are able to persist and propagate in the environment, potentially occurring next generation effects may pose specific challenges for risk assessment (Bauer‐Panskus et al. 2020). As described by Breckling and Schmidt (2015), in this context, risk assessment has to consider the genetic modification at the molecular level as well as the connected cause–effect chains to higher levels of the cells, the organisms, the populations, the ecosystems, and the landscapes. Next generation effects in ERA are not completely new but deserve much more attention in the context of gene drive organisms, which will typically take dozens of generations to show the intended effects in the target population (Oye et al. 2014) and, due to changes in pattern of inheritance, have to be regarded as being invasive in most cases (Noble et al. 2018; Frieß et al. 2019). Therefore, especially in the case of gene drive organisms, the spatial and temporal dimension will become decisive in the overall risk assessment. In replacement drives, the goal is to introduce an artificial genetic element into natural populations so that it persists over a longer period of time. In the long term, the probability of unintended changes in biological characteristics is higher in replacement drives than in suppression drives.

The basic challenge for risk assessment in this context is how regulatory decisions can be made in the face of substantial nonknowledge. To solve this problem, we propose applying cut‐off criteria similar to those applied in the EU regulation of chemicals. To define these cut‐off criteria within the regulatory decision‐making process on GE organisms, a new step in the risk assessment of GE organisms is recommended, that is, spatiotemporal controllability. This can be seen as a cut‐off criterion analogous to PBT and vPvB that are anchored in the EU regulation of chemical substances (EC 2006). The approach uses specific “knowns” to decide upon “known unknowns” (such as next generation effects and genomic × environmental actions). It is assumed that the criteria used to assess spatiotemporal controllability can inform regulatory decision making even in the light of major uncertainties emerging from the spatial and temporal dimension: Even if the overall risk assessment cannot be concluded, the assessment of spatiotemporal controllability is likely to provide reliable results. It is an advantage that the introduction of this additional step in ERA would not require any change in EU law, but has to be understood as an implementation of current GMO regulation, framed by Directive 2001/18/EC (EC 2001).

It is important to note that the assessment of spatiotemporal controllability as suggested is not an assessment of a specific risk per se, but it can contribute to the overall conclusiveness of risk assessment. Our approach is illustrated in the case study of GE olive flies carrying a gene drive that renders lethality to female offspring. Our case study indicates that the assessment of spatiotemporal controllability produces results that are meaningful, comparable, and allow the application of cut‐off criteria within the process of risk assessment. To test our approach, we also compared spatiotemporal controllability in examples such as GE oilseed rape and GE maize. We obtained differing results, which are dependent on the regional specificity of the receiving environments as well as on the biological characteristics of the plants (data not shown).

Similar results that can be scaled and compared may also be expected for future gene drives. However, the schematic and partially hypothetical case study in the present paper does not present all the relevant data that would be included in a real dossier for application for environmental releases. The proposed approach can be considered flexible enough to be improved by adding further criteria and new data. It can be employed for applications for environmental releases, regardless of consent for experimental field trials or commercial release.

The criterion as suggested can also be informative for upstream processes and thereby generate more clarity and certainty at an early stage of research and development because many researchers currently developing gene drive applications are already aware of the problem of spatial and temporal complexity. In this context, our approach may also be useful in combination with the concept of Quantitative Risk Assessment for Synthetic Biology Products as proposed by Trump et al. (2018). This concept aims to comprehensively address all hazards with catastrophic potential at an early stage of research and development, leaving it to a more case‐specific risk assessment to deal with the other remaining issues. Within this concept, the criterion as proposed can also be applied in other regulatory systems, which so far do not have a precautionary and centralized GMO regulation, as is the case in the EU (Bar‐Yam et al. 2012; Greer and Trump 2019), and follow the perspective of prospective technology assessment (Frieß et al. 2019).

It should be kept in mind that although spatiotemporal controllability is relevant for the implementation of the PP, there are other reasons why risk managers should determine relevant cut‐off criteria. According to the Cartagena Protocol on Biosafety (CBD 2000), if GE organisms spontaneously cross borders (unintentional transboundary movement), their release has to be reported immediately in order to enable appropriate responses, including emergency measures, in the interest of minimizing any adverse effects on biodiversity and risks to human health.

Further, in the overall decision‐making process on potential releases of GE gene drive organisms, other stakeholders apart from scientific institutions also need to be taken into account (see Schmidt et al. 2009; Bar‐Yam et al. 2012; Engelhard et al. 2016; Cummings and Kuzma 2017; Lunshof and Birnbaum 2017; Trump et al. 2018). Especially ethical and cultural aspects need to be considered and should not be left out of the process because, for example, gene drives have the potential to substantially interfere with biodiversity or eradicate natural populations of the targeted species.

## CONCLUSIONS

It has been shown that the risk assessment of intended environmental releases of GE organisms linked to self‐propagation of artificial genetic elements over several generations will suffer from major uncertainties and unknowns emerging from potential next generation effects. Therefore, the spatial and temporal complexity will be substantially increased. These problems apply not exclusively to GE gene drive organisms, but also to the release of GE organisms and are, for example, also being discussed in the field of synthetic biology. However, for the assessment of gene drives, which typically show invasive characteristics and will take dozens of generations to render the effects as intended, the issue of spatial and temporal complexity becomes a matter of much greater urgency.

It can be assumed that at a certain point in the dissolution of spatial and temporal boundaries, it will become necessary to apply cut‐off criteria and halt the approval process. This means that risk assessors and risk managers face the problem of how to come to robust conclusions and reliable decisions within the approval process that also give substantial weight to the PP. Within the EU regulatory framework, we propose the introduction of the cut‐off criterion of spatiotemporal controllability as an additional step in ERA. This concept can be used to delineate some of the boundaries between knowns and unknowns considered to be crucial. It will foster the robustness of risk assessment and can substantially benefit the reliability of decision making within approval processes. The introduction of spatiotemporal controllability as an additional step within ERA does not require any change in EU law; it can be regarded as the implementation of the PP as foreseen by EU Directive 2001/18/EC (EC 2001). The concept of cut‐off criteria might also be applied in prospective technology assessment and might thereby become especially valuable in regulatory systems outside the EU, which so far do not have an established precautionary and centralized approach in GMO regulation. In this context, the cut‐off criterion as proposed can help to address hazards with catastrophic potential at an early stage of research and development.

## Data Availability

No new data were generated for this manuscript, and all data used are available in the cited references.
